# Group A *Streptococcus* virulence factors genes in north India & their association with *emm* type in pharyngitis

**Published:** 2011-01

**Authors:** V. Dhanda, H. Vohra, R. Kumar

**Affiliations:** * *School of Public Health, Postgraduate Institute of Medical Education & Research, Chandigarh, India*; ** *Department of Experimental Medicine & Biotechnology, Postgraduate Institute of Medical Education & Research, Chandigarh, India*

**Keywords:** *emm* types, GAS, virulence factors

## Abstract

**Background & objectives:**

Group A streptococcal (GAS) pharyngitis, especially among children, leads to high prevalence of rheumatic fever (RF)/rheumatic heart disease (RHD) in India, as compared to the western world where invasive diseases are common. GAS encodes numerous virulence factors that cause diseases by exhibiting extraordinary biological diversity. Hence, we studied the virulence factors genes of GAS isolated from the throat of children with pharyngitis and also asymptomatic carriers.

**Methods:**

Fifty GAS isolates cultured from throats of north Indian children aged 5-15 yr with mild pharyngitis (20), severe pharyngitis (24) and asymptomatic pharyngeal carriers (6), during 2000-2003 along with reference M1 strain were *emm* typed and characterized for virulence factors genes by PCR. The presence of virulence factors was also checked for their association with *emm* type in pharyngitis.

**Results:**

Twenty *emm* types, six sequence types, and one non-typeable strain were found circulating in north India. The five most prevalent types were *emm* 74 (12%), 11 & StI129 (8% each) and *emm* 68 and NS292 (6% each). The spe B gene was found to be significantly higher (*P*=0.0007) in opacity factor (OF) negative isolates. *emm* 3, 11, 77, 86, 87, 109 and StI129 showed maximum virulence factors genes.

**Interpretation & conclusions:**

GAS isolates collected from throats of children from north India possess highly virulent antigens. This study also supports concept of isolate-associated virulence rather than type relatedness.

Group A *Streptococcus* (GAS, *Streptococcus* pyogenes) shows an incredible history of changing disease pattern^1^ with numerous cell surface associated and secretary factors required for adherence and colonization of the host at various sites, destruction of the tissues for facilitating the spread, and other systemic effects causing autoimmune complications^2^. Proteomic analysis of GAS recently identified 79 proteins on its surface and 21 cytoplasmic proteins^3^. Streptococcal adherence to host pharyngeal epithelium is the basic step in colonization. Environmental conditions, cell density and growth phase are known to influence expression of virulence factors^4^,^5^.

Globally, GAS has emerged as a highly variable organism. High prevalence of rheumatic fever (RF)/rheumatic heart disease (RHD) in India in comparison to invasive diseases from developed nations and heterogeneity of GAS *emm* types have strengthened the need for looking into their virulence potential. The association of virulence factors, whether protein-binding or exotoxin with pharyngitis that ultimately leads to RF/RHD is poorly understood in Indian scenario. Therefore, an attempt was made to study the distribution of genes coding such factors to facilitate understanding of bacterial pathogenesis in GAS isolates from throat carriers and pharyngitis cases.

## Material & Methods

A total of 50 clinical GAS isolates, collected from children aged 5-15 yr from Raipur Rani block of Panchkula, district Haryana and Government Medical College, Chandigarh (November 2000 to July 2003), identified by a Streptex Murex kit (Remel Europe Ltd, UK) and one M1 reference GAS strain were used in the present study. GAS isolates were categorized on the basis of severity of signs and symptoms of GAS pharyngitis into three groups: severe pharyngitis (n=24); mild pharyngitis (n=20) and no pharyngitis (n=6).

The opacity factor (OF) of these isolates was determined in a 96 well microplate^6^ and the results were read at 450 nm with an ELISA microplate reader (Tecan Austria, GmbH). Twenty seven GAS isolates (54%) were OF negative; 54 per cent in severe, 50 per cent in mild pharyngitis and 66 per cent in those without pharyngitis, *i.e*., in almost equal proportion among the three clinical categories.

Genomic DNA was isolated by Qiagen kit (Dneasy Tissue Kit, Qiagen GmbH, Germany) as per manufacturer’s instructions and preparations of OD_260_/OD_280_ >1.8 were used as template for PCR of different virulence factors. *emm* gene was identified using specific primers and following standardized PCR thermo cycling conditions^7^. PCR products were checked on 0.8 per cent gel, analyzed for yield and then purified by QIAGEN PCR purification kit. The *emm* gene sequencing was done in an ABI 377 Automated Sequencer as per manufacturer’s instructions (Applied Biosystems, USA) and then gene sequence was searched for homology at CDC website as described earlier^7^.

The isolated DNA was used for amplifying nucleotide sequences corresponding to the regions of selected virulence factors: *slo, ska, pbp, spe A, spe B, spe C, prtf, sfb, fbp-54, scp* and *sic* by PCR. The PCR reaction mixture with specific primer of different virulence factors was prepared. All reagents were procured from Roche Molecular Biochemicals (Boehringer Manheim, Germany). PCR products were electrophoresed through 1.5 per cent agarose gel and separated bands were visualized under UV trans-illuminator after ethidium bromide staining (Sigma Chemicals Co, USA) and analyzed using Video Gel Documentation System (Imagemaster gel documentation system, M/S Bio-Rad Laboratories Pvt Ltd, Australia). EPI-6 (Epi Info Version 6) statcalc software was used for data analysis. Chi square test was applied and *P*<0.05 was considered to be statistically significant.

## Results & Discussion

Significant diversity of *emm* types was observed. A total of 27 *emm* types; 20 known *emm* types, 6 sequence-types and a novel M non typeable strain were identified. The five most prevalent types were: *emm* 74 (12%), 11 and StI129 (8% each) and *emm* 68 and NS292 (6% each). Majority (65%, 13/20) of the isolates belonging to the five most prevalent types were OF negative; all the four isolates of *emm*11; 5 out of the 6 *emm*74 isolates and 3 out of the 4 StI129 isolates were OF negative, however, all the three isolates of *emm* 68 and two out of the three isolates of NS292 were OF positive. Amongst the less prevalent types *emm* 3, 28, 77, 49 and 74 were OF negative, and *emm* 2.1, 60, 68, 75, 81, 109 were OF positive (Table).

**Table:** Distribution of virulence factor genes in group A *Streptococcus emm* types

Sr. No.	*emm* type	OF	*slo*	*ska*	*pbp*	*spe A*	*spe B*	*spe C*	*fbp-54*	*sfb*	*prtf*	*scp*
Presence of virulence factor genes												
	Reference strain M1	-	+	+	+	+	+	-	+	-	-	+
	Severe Pharyngitis											
1	AAB5113	-	+	+	+	+	+	-	+	-	+	+
2	Sp11014/VT-15	+	+	+	+	-	+	-	+	-	+	+
3	Sp11014/VT-15	-	+	+	+	-	+	-	+	+	+	+
4	StI129	+	+	+	+	-	+	+	+	+	+	+
5	74	-	+	+	+	-	+	-	+	+	+	+
6	87	+	+	+	+	+	+	-	+	+	+	+
7	3	-	+	+	+	+	+	-	+	+	+	+
8	NS292	+	+	+	+	+	+	-	+	+	+	-
9	74	-	+	+	+	-	+	+	+	+	-	+
10	AAL28405	-	+	+	+	+	+	+	+	-	+	+
11	109	+	+	+	+	-	+	-	+	+	+	+
12	74	-	+	+	-	-	+	-	+	+	+	-
13	11	-	+	+	+	-	-	-	+	+	+	+
14	81	+	+	+	+	-	+	-	+	-	+	+
15	71	-	+	+	+	-	+	-	+	+	+	+
16	49	+	+	+	+	+	+	-	+	-	+	+
17	68	+	-	+	+	-	+	+	+	+	+	+
18	102.1	-	+	+	+	-	+	+	+	-	-	+
19	3	-	+	+	+	+	+	-	+	+	-	+
20	86	+	+	+	+	+	+	-	+	+	+	+
21	77	-	+	+	+	+	+	-	+	+	+	+
22	74	+	+	+	+	+	-	-	-	-	+	+
23	StI129	-	+	+	+	+	-	+	+	-	-	+
24	60	+	+	-	+	-	+	-	+	-	+	+
	Mild Pharyngitis											
25	68	+	+	+	+	-	+	+	+	-	-	+
26	28	-	+	+	-	-	-	-	-	-	+	-
27	TP-c2135	-	+	+	+	-	+	+	+	+	-	+
28	89	+	+	+	+	+	+	-	+	-	-	+
29	75	+	+	+	-	-	+	-	-	-	-	-
30	74	-	+	+	+	-	+	-	+	-	-	+
31	11	-	+	+	+	+	-	+	+	+	-	+
32	77	-	+	+	+	-	+	-	+	-	+	+
33	11	-	+	+	+	+	+	+	+	-	+	+
34	NS292	+	+	+	+	-	+	-	+	-	+	+
35	74	-	+	+	+	+	+	-	+	+	-	+
36	2.1	+	+	+	+	-	+	+	+	+	-	+
37	11	-	+	+	+	-	+	-	+	+	+	+
38	49	-	-	+	+	+	+	-	+	-	-	+
39	65	+	+	+	+	-	+	-	+	+	+	+
40	81	+	+	+	+	-	+	-	+	+	+	+
41	93	-	+	+	-	+	+	-	+	-	+	+
42	42	+	+	+	+	+	+	+	+	-	+	+
43	SP10741/Allele75	+	+	+	+	+	-	-	-	+	-	+
44	71	+	+	+	-	-	-	-	+	-	+	-
	Asymptomatic											
45	109	+	+	+	+	+	+	-	+	+	+	+
46	68	+	+	+	+	-	+	-	-	-	+	+
47	stI129	-	+	+	-	+	-	-	-	-	+	+
48	AAL28405	-	+	+	-	-	-	-	-	-	-	-
49	NS292	-	+	+	-	-	-	-	-	-	-	-
50	StI129	-	+	+	-	-	-	-	-	-	-	-
Frequency of Clinical Isolates												
	%	54	96	98	82	42	78	24	82	50	72	84

Percentage of isolates with following genotype = No. positive/No. tested (50) X 100. OF - opacity factor, *slo* - streptolysin, *ska* - streptokinase, *pbp* - plasminogen binding protein, *spe A, spe B* & *spe C* - streptococcus pyogenic exotoxin A, B , respectively, *fbp-54* - fibronectin binding protein, *sfb* - streptococcal fibronectin binding protein, *prtf* - fibronectin binding protein F and *scp*- C_5a_ peptidase. + presence, - absence

The amplification for *slo, ska, sfb, fbp-54, prtf, spe A, spe B, spe C, scp* and *pbp* genes when checked on agarose gel was found to be of <500, ~400, ~1400, 1500, <500, ~900, 1400, 1000, <1000 and 1200-1500 base pair in size, respectively. None of the isolates were positive for *sic* gene. When the OF production was compared with the prevalence of virulence genes, spe B was found to be statistically more common in OF negative isolates (*p*=0.0007), indicating its role in the pathogenesis.

The presence of *slo* and *ska* was comparable to the previous studies^8^,^9^. Most of the GAS strains encode *spe B* while *spe A* and *spe C* occur less frequently. The strains associated with severe infections have been shown to produce spe A toxin^9^,^10^. Although there are few reports on the occurrence of scarlet fever and toxic shock syndrome from India, comparatively high frequency of *spe A* (42%) from non-invasive pharyngitis cases was found from worldwide^9^,^11^. In the past Nandi *et al*^12^ demonstrated low frequency of spe A gene (8.3%) within Indian isolates, indicating their less virulent nature, but the present study gives an indication that virulent strains are circulating within the Indian community. Such strains which have ability to produce specific exotoxins, in the absence of type specific immunity in a population, may lead to a community outbreak of streptococcal infections. *spe B* gene is assumed to be chromosomal encoded and highly conserved. Tyler *et al*^9^ showed *spe B* gene in all or majority of the GAS isolates, however all strains may not have it. Comparatively low frequency (78%) of *spe B* gene was found in our study than 93.3 per cent observed in the past from the same region^12^ and 99.3 per cent from Canada^9^ indicating that it might not have been retained during the course of evolutionary events that have also generated extensive genomic diversity in our population. The gene frequency for spe C (24%) was comparable with earlier study^9^,^11^. Almost all strains of GAS possess *scp* gene, similar to earlier report^13^. *Fbp-54* gene has been reported in all clinical isolates, however its expression differs quantitatively^14^, in present study it was 82 per cent. Our frequency of prtf gene (72%) was similar to observations from Japan with prevalence of 77.3 per cent^15^. The *sfb* gene proportion was also comparable to published data with the frequency ranging between 50-70 per cent in all clinical isolates^16^. We could not find any GAS isolate positive for *sic* gene in contrast to closely related *sic* (crs) and distantly related *sic* (drs) genes encoding this protein reported recently from India and Japan^15^,^17^. It has been reported that all M1 and M57 strains have *sic* (crs); and all M12 and M55 strains have *sic* (drs) and hence express the respective proteins^18^. We could not isolate M1, 12, 55 and 57 types in our sample that could be well associated with the absence of sic gene from our GAS isolates.

The reference M1 strain possessed maximum virulence factors tested except *spe C, sfb* and *prtf*. The most commonly and least found genes were *ska* and *spe C* respectively. The most predominant *emm* types had almost all the virulence factors except *sic: emm* 11, 42 and AAL28405 lacked *sfb* gene; StI129 lacked spe A gene; and *emm* 87, 3, 86, 77 and 109 did not have spe C gene. AAL28405, *emm* 42 and 11 had genes for three exotoxins, and *emm* 11, 65, 81, 74, 87, 3, 109, 74, 11, 71, 68, 86, 77, NS292, StI129 and Sp11014/VT-15 possessed all the three fibronectin binding protein (FBP) genes. Hence *emm* 3, 11, 77, 86, 87, 109 and StI129 showed maximum number of virulence factors. The most prevalent five types (20 isolates) showed frequency of *slo, ska, spe A, spe B, spe C, fbp-54, sfb, prtf, pbp* and *scp* virulence factors genes in 95, 100, 35, 65, 35, 75, 50, 70, 80 and 80 per cent isolates respectively.

Varied genotypic combinations of the selected virulence factors were obtained in the GAS isolated from patients with throat infection of different severity (Table). *Slo, ska, pbp, fbp54, prtf* and *scp* encoding for major adherence/attachment factors were found in nearly 90 per cent of the severe pharyngitis cases whereas asymptomatic cases predominantly showed *slo, ska* and *prtf*. Significant association (*P*<0.05) was observed for the virulence factor genes in the three clinical pharyngitis categories (Fig.). The frequency of genes *pbp, spe B* and *fbp-54* was significantly high in severe pharyngitis cases in comparison to asymptomatic cases (*P*<0.05). Significantly higher frequency of *prtf* (*P*=0.04) was seen in severe pharyngitis as compared to the ones isolated from mild pharyngitis cases. The frequency of *fbp-54* was significantly high (*P*=0.01) in mild pharyngitis compared to asymptomatic cases.

**Fig.**
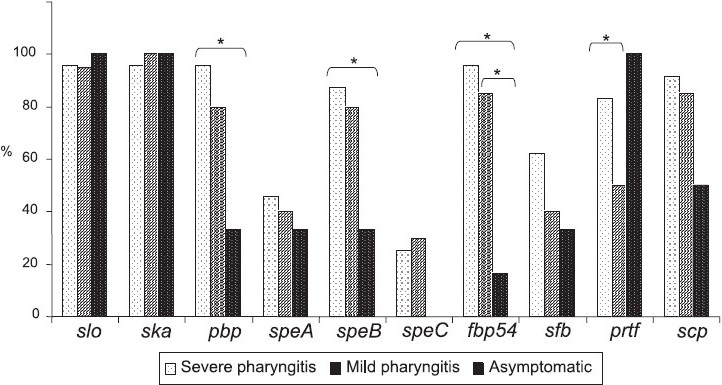
Virulence factors genes and their association with severity of pharyngitis. *Significant (*P*<0.05).

Since *slo, ska, spe A, sfb* and *scp* were present in all the throat isolates studied, their presence or absence may not be associated with increased virulence. Similar to our study for FBP genes particularly *prtf*, no significant difference in their distribution in pharyngitis isolates and asymptomatic carriers was observed earlier^19^. All the GAS isolates from asymptomatic cases carried the *prtf* gene, almost similar to the previous findings. High proportion of *fbp-54* was described in GAS from asymptomatic carriers, however, it was quite low in our study (Fig.). Hence, GAS might recruit different FBPs genes for different purposes. Individual contributions of *spe* genes that elicit potent inflammatory responses is currently unclear, but is reported in invasive GAS diseases^20^. High frequency of spe A and its production associated with severity has been predicted^10^, a case different than *slo, spe B* and *spe C*^9^. The frequency of *spe A* gene (43%) in pharyngitis or RF/RHD GAS isolates in the present study was higher as compared to reports from India and elsewhere (6 to 25%)^9^,^12^; but quite similar to the reports from severe infections (42.9%)^12^, which is a matter of concern. *spe B* (84%) in GAS isolated from pharyngitis was in agreement with the previous findings (86.5%) from the same region^12^. Its frequency was less (33%) in asymptomatic cases, justifying its role in severe streptococcal diseases. *Spe C* was present in 25 per cent GAS isolates from severe pharyngitis cases and was absent in asymptomatic cases as compared to 55.4 and 65.8 per cent reported earlier^11^. No association of asymptomatic GAS with *spe C* gene or its production *in vitro* has been reported. The fact that almost all GAS types possess *scp* gene is a reason to believe that specific cleavage of C5a chemotaxin contributes to the virulence of these streptococci^13^.

On comparing OF negative and positive isolates for throat infection severity in terms of their virulence factors, significant differences (*P*<0.05) were recorded for *pbp, spe B, fbp-54, sfb, scp* in OF negative; and *prtf* in OF positive isolates respectively, suggesting heterogeneous distribution of virulence factors in OF negative GAS isolates.

This study demonstrated different *emm* types in GAS isolates, with varied genotypic potential. GAS isolates collected from northern India possessed highly virulent antigens. This study also supports concept of isolate-associated virulence rather than virulence broadly related to a given serotype.
